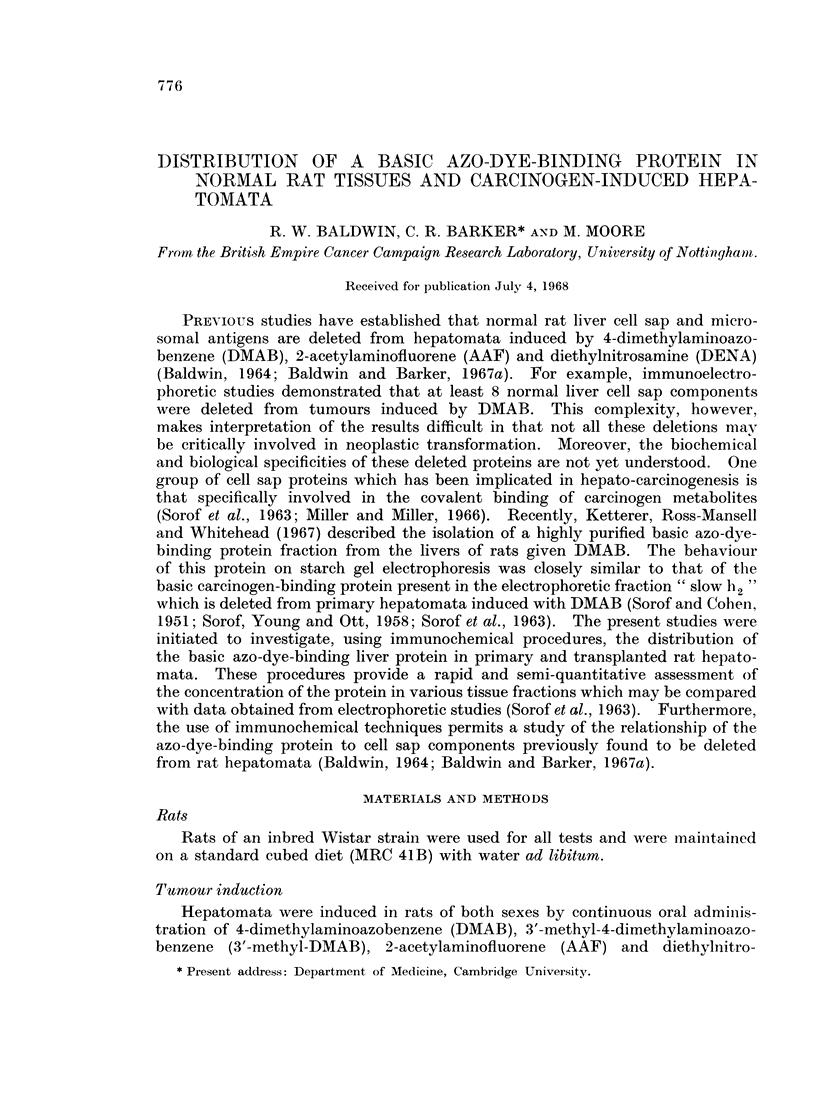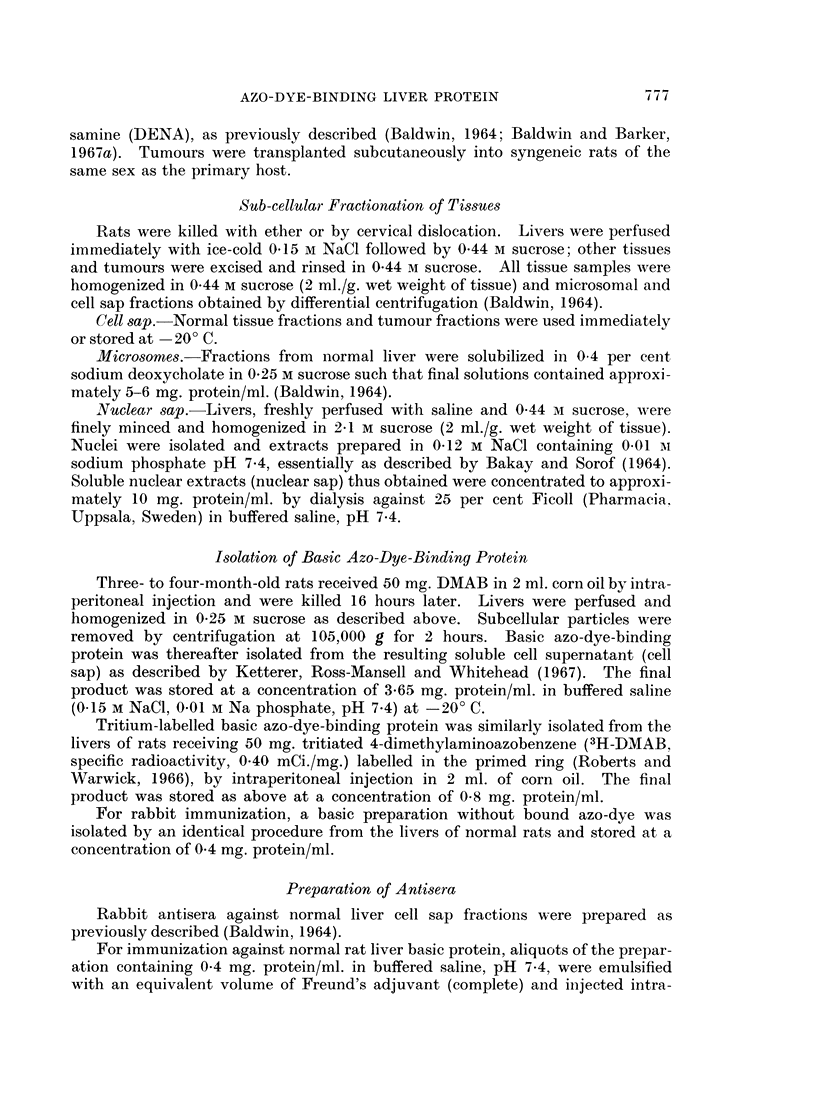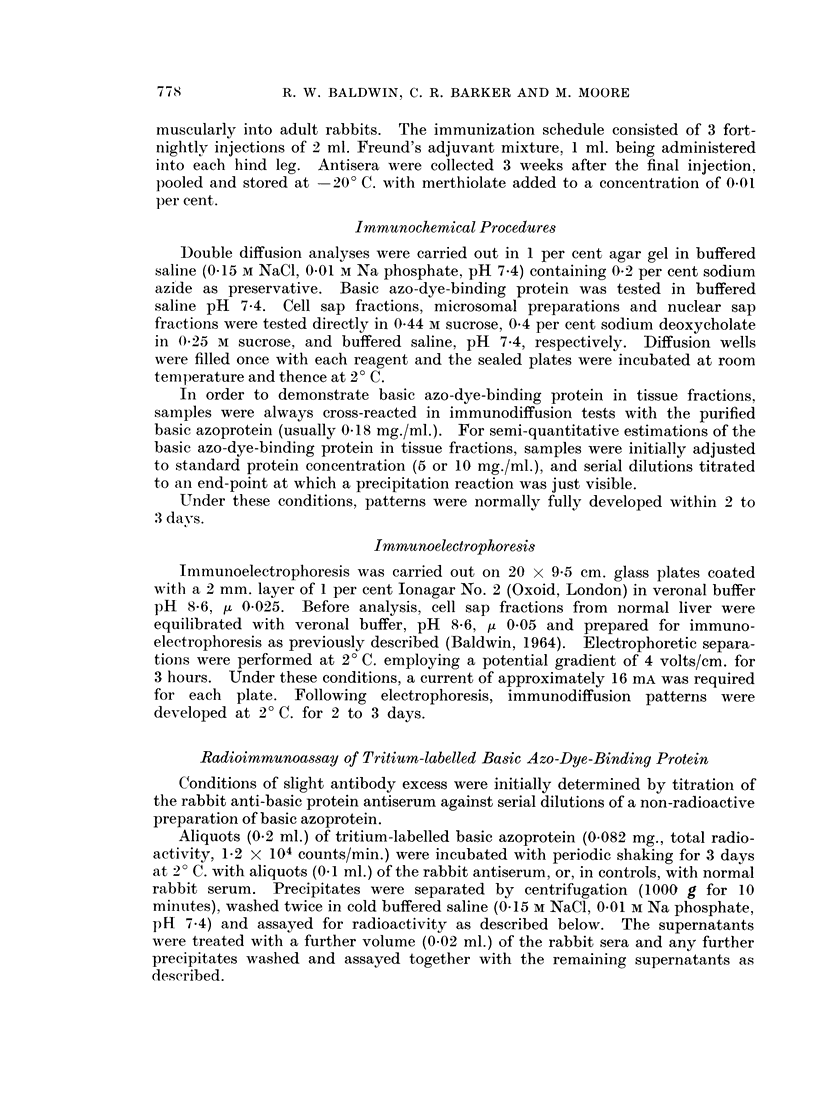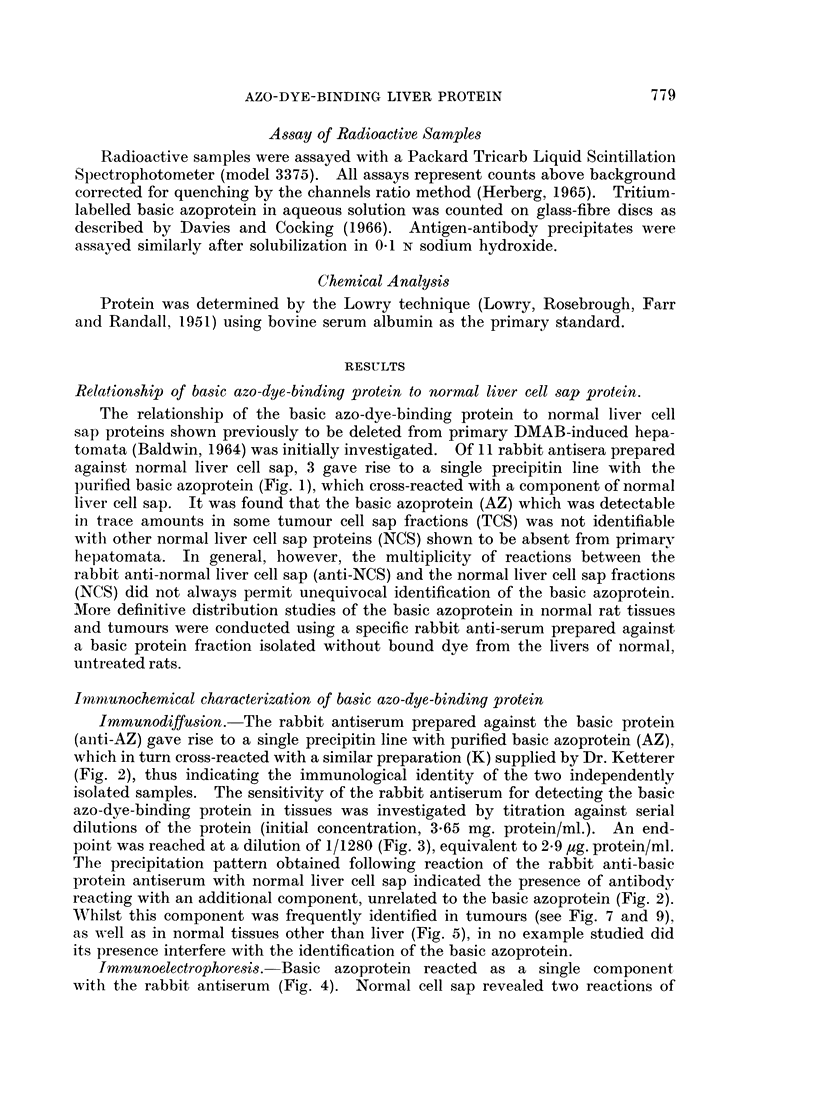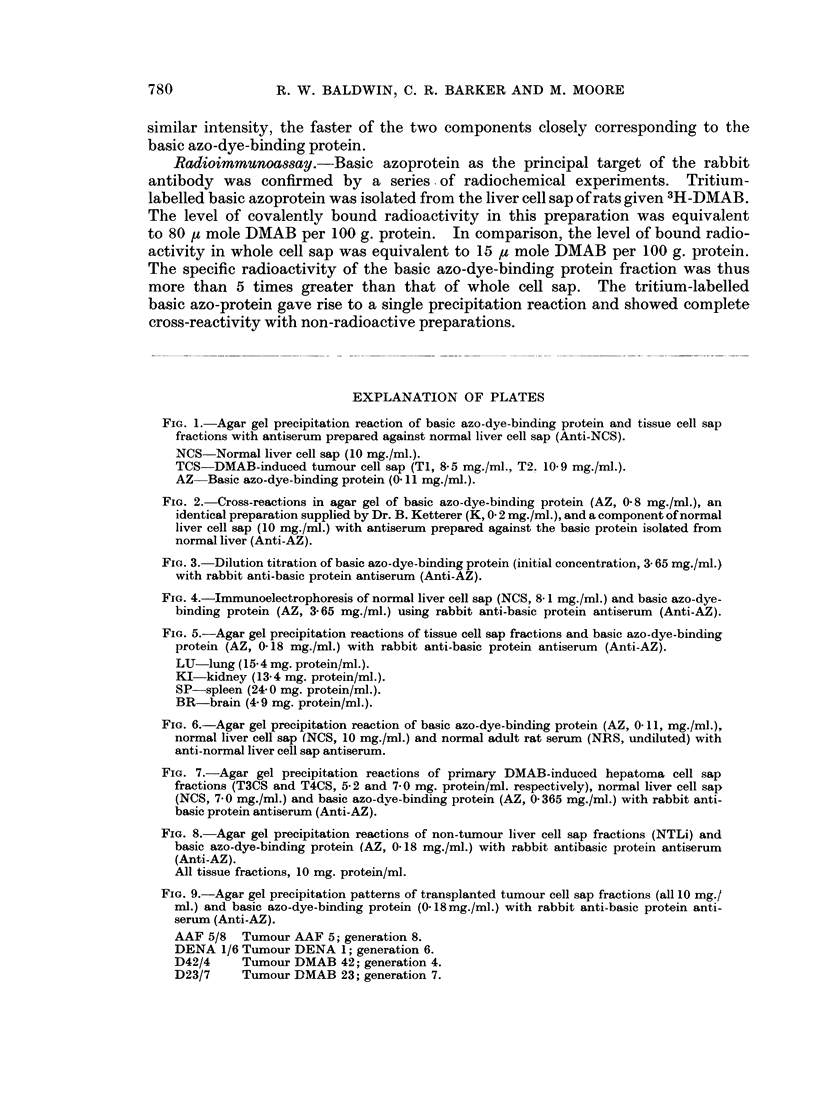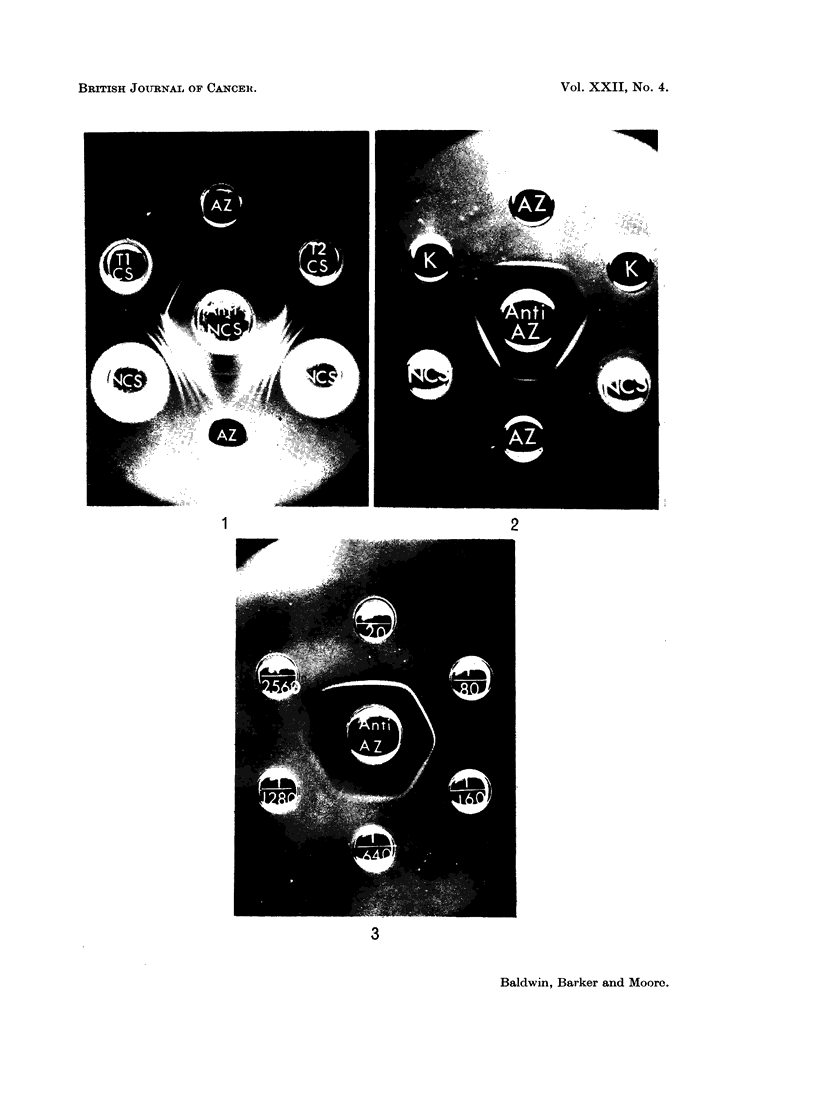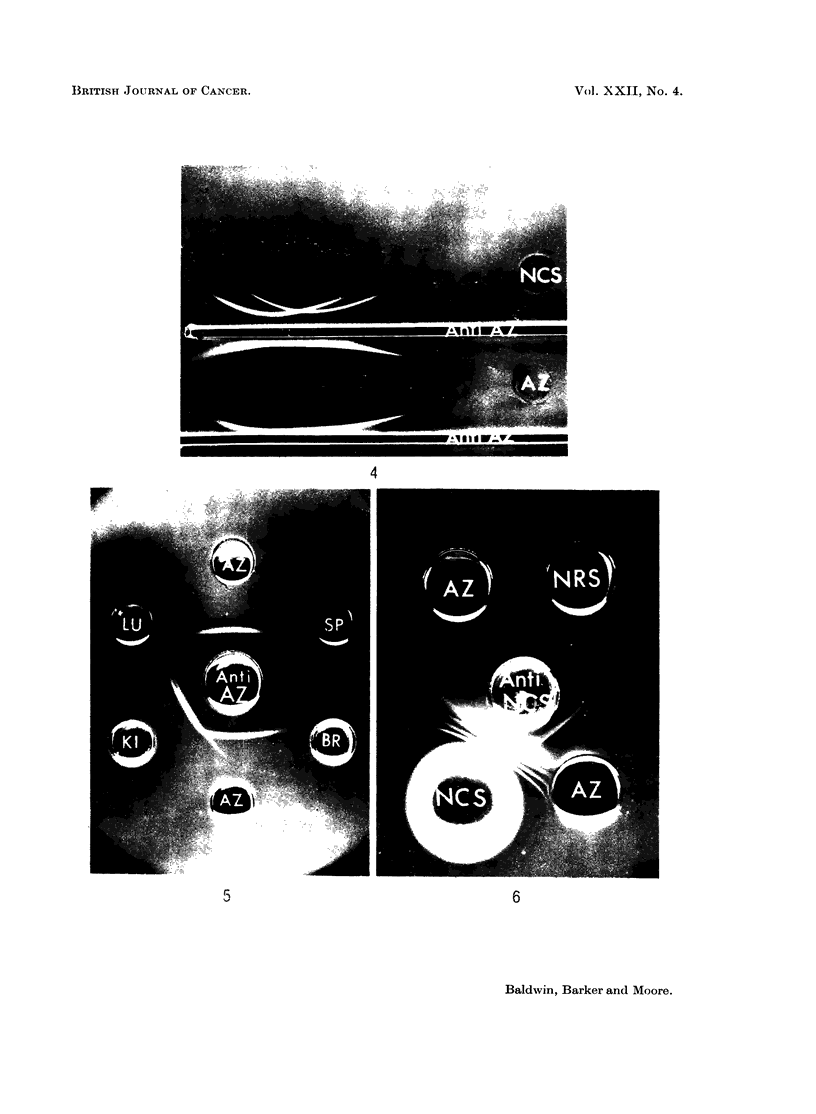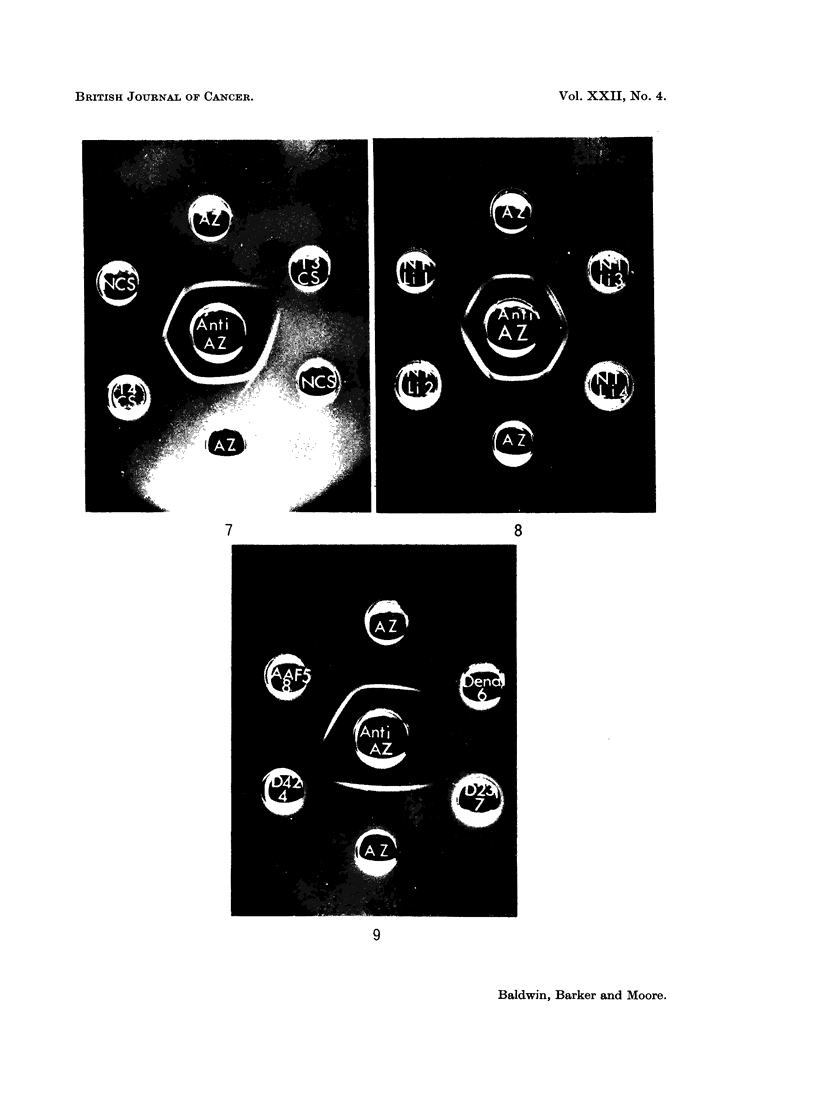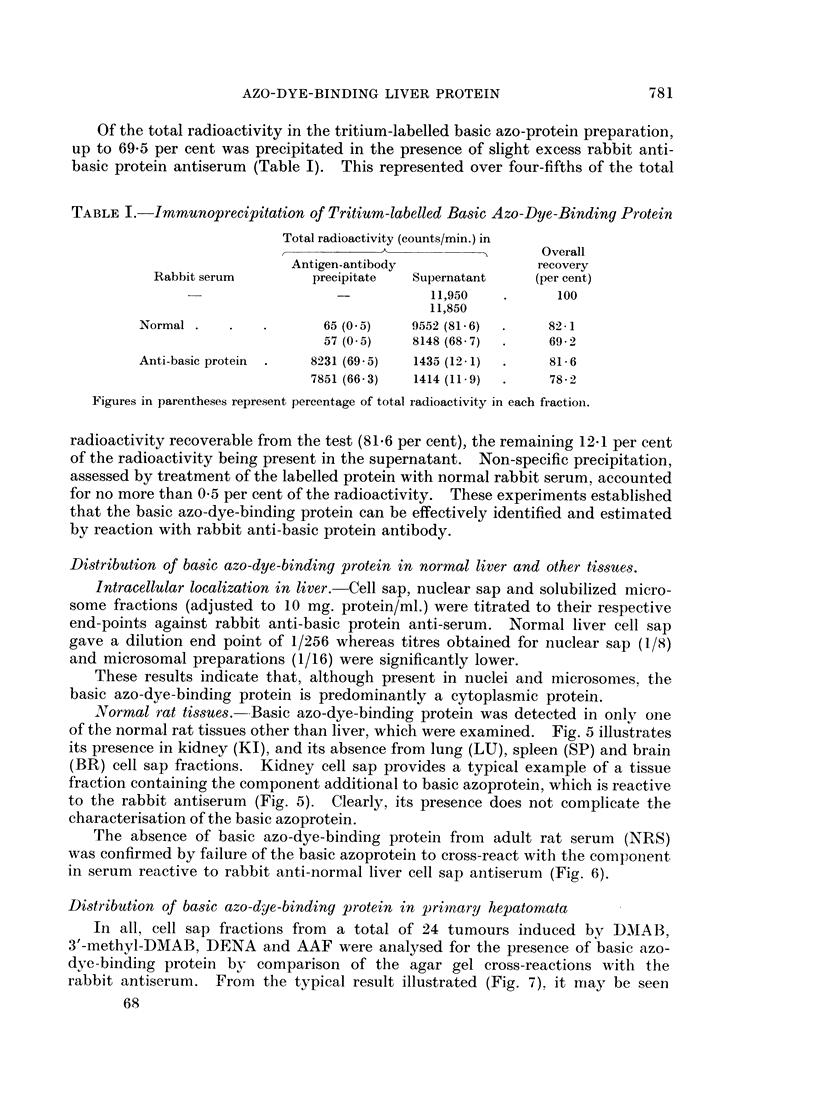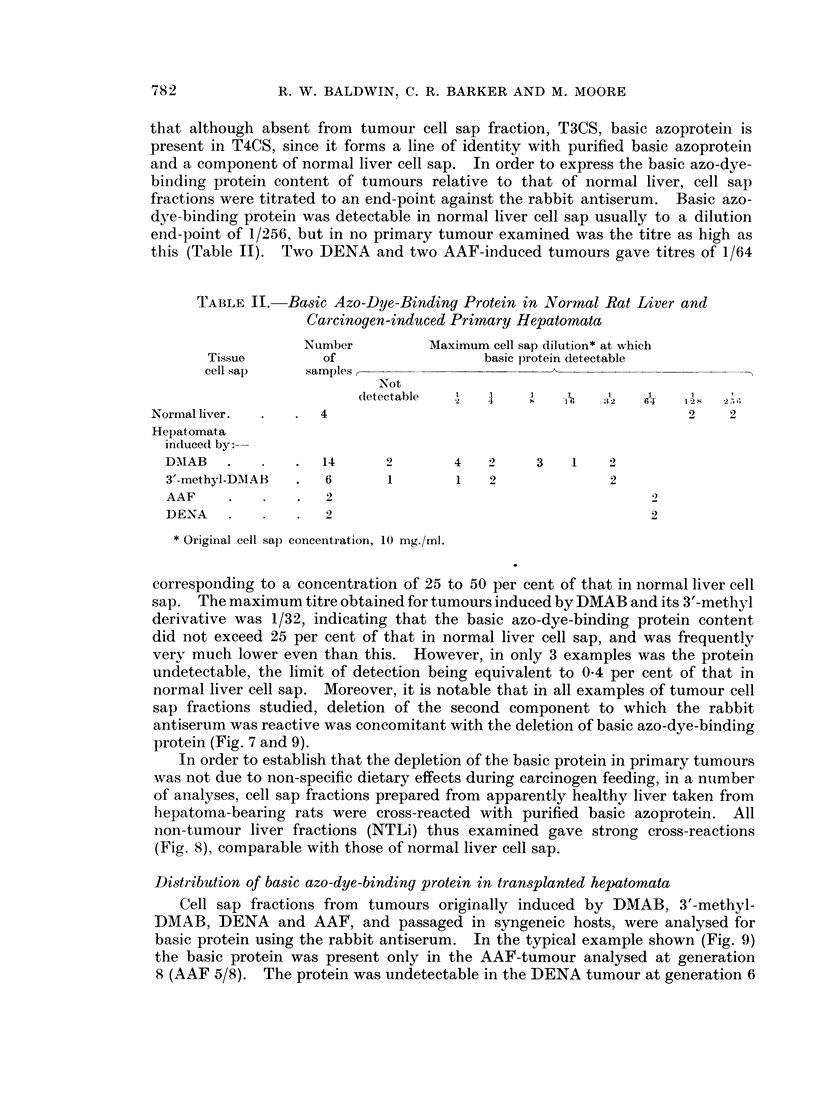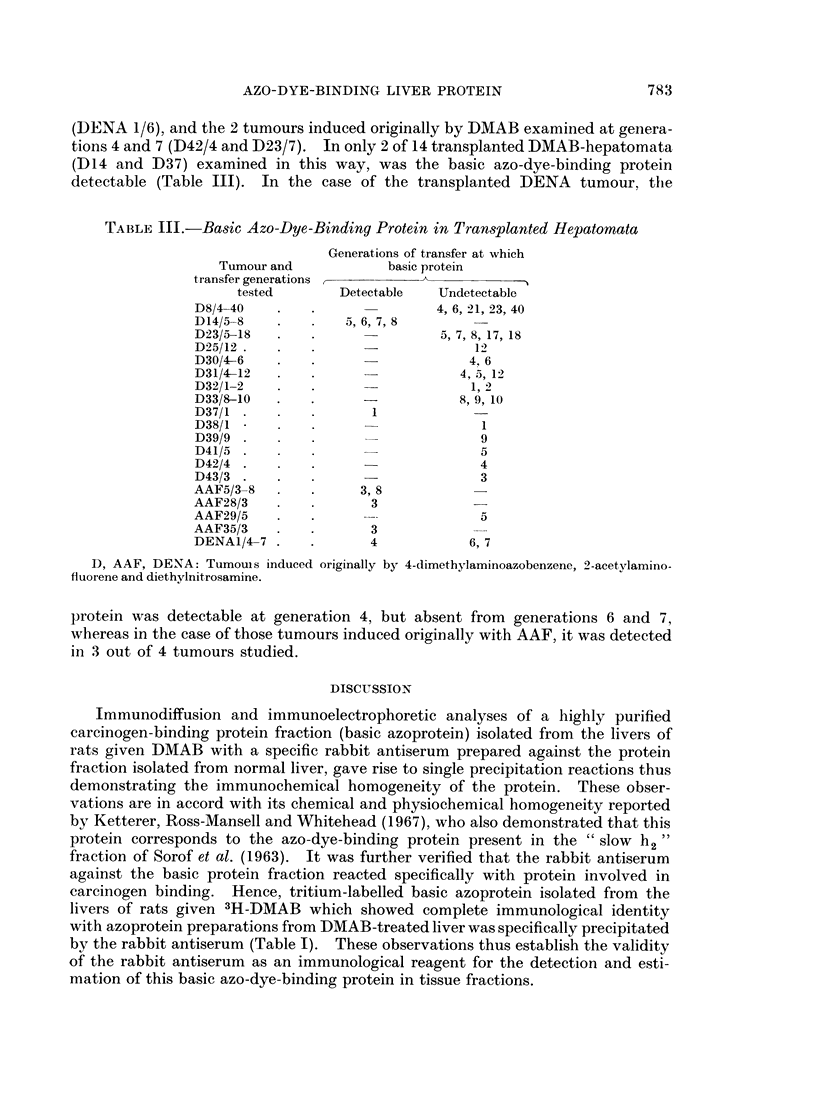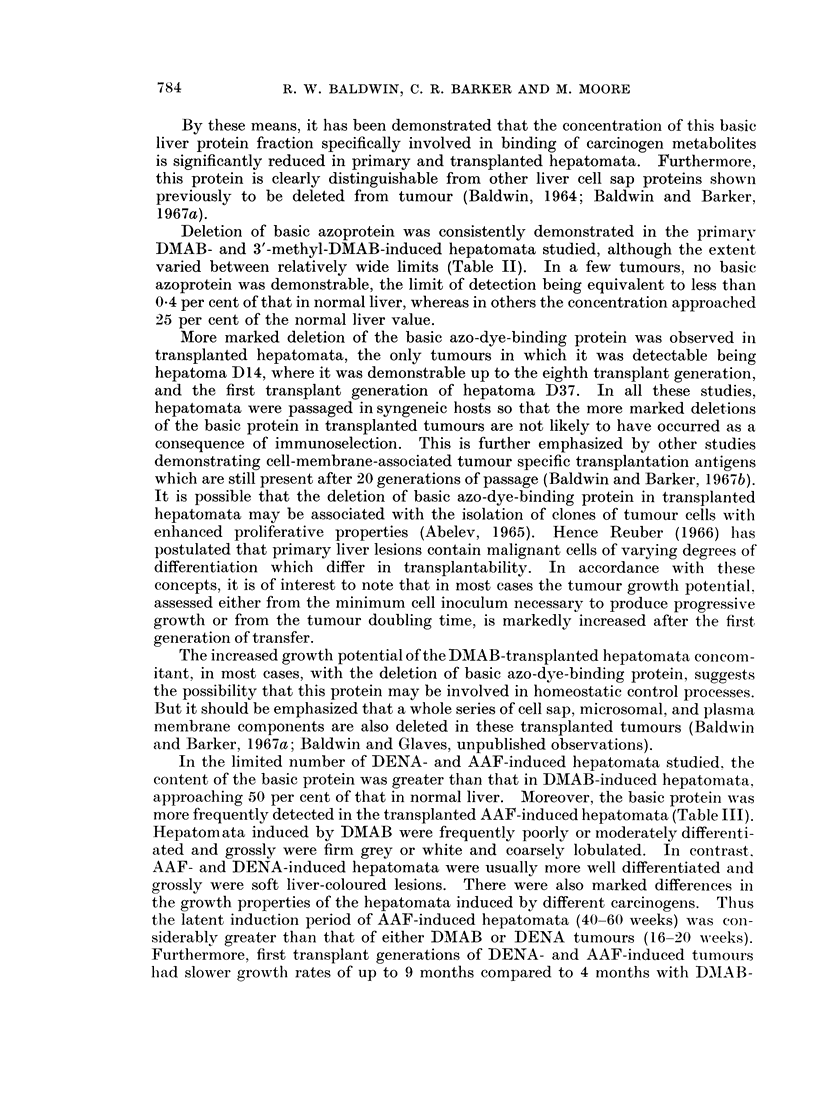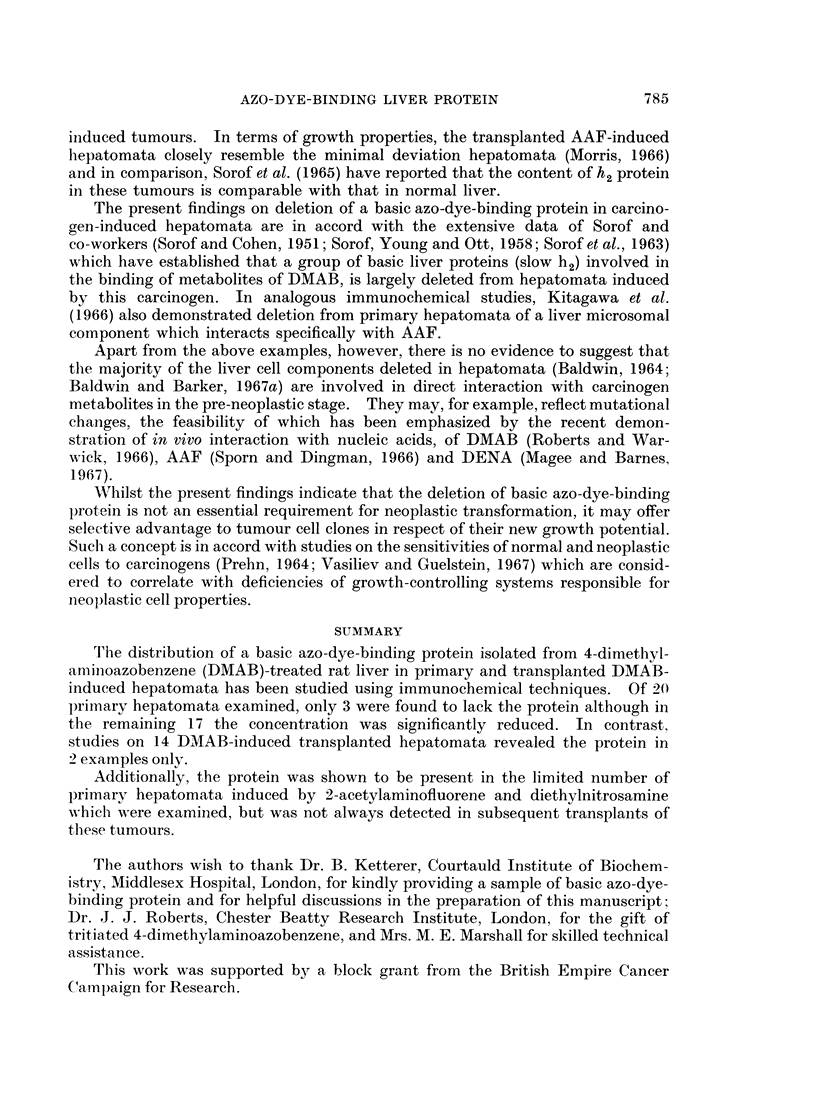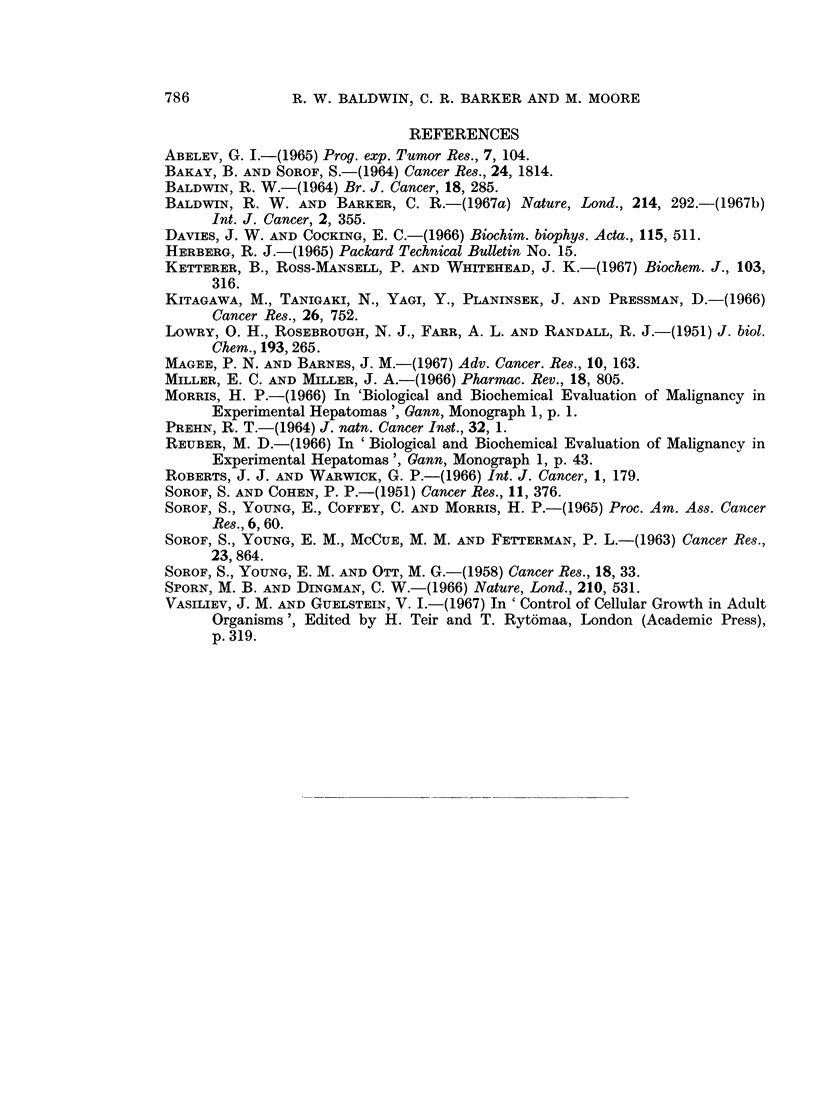# Distribution of a basic azo-dye-binding protein in normal rat tissues anc carcinogen-induced hepatomata.

**DOI:** 10.1038/bjc.1968.92

**Published:** 1968-12

**Authors:** R. W. Baldwin, C. R. Barker, M. Moore

## Abstract

**Images:**


### DISTRIBUTION OF A BASIC AZO-DYE-BINDING PROTEIN IN NORMAL RAT TISSUES AND CARCINOGEN-INDUCED HEPATOMATA

R. W. BALDWIN, C. R. BARKER* AND M. MOORE

*From the British Empire Cancer Campaign Research Laboratory, University of Nottingham.*

Received for publication July 4, 1968

PREVIOUS studies have established that normal rat liver cell sap and microsomal antigens are deleted from hepatomata induced by 4-dimethylaminoazobenzene (DMAB), 2-acetylaminofluorene (AAF) and diethylnitrosamine (DENA) (Baldwin, 1964; Baldwin and Barker, 1967*a*). For example, immunoelectrophoretic studies demonstrated that at least 8 normal liver cell sap components were deleted from tumours induced by DMAB. This complexity, however, makes interpretation of the results difficult in that not all these deletions may be critically involved in neoplastic transformation. Moreover, the biochemical and biological specificities of these deleted proteins are not yet understood. One group of cell sap proteins which has been implicated in hepato-carcinogenesis is that specifically involved in the covalent binding of carcinogen metabolites (Sorof *et al.*, 1963; Miller and Miller, 1966). Recently, Ketterer, Ross-Mansell and Whitehead (1967) described the isolation of a highly purified basic azo-dye-binding protein fraction from the livers of rats given DMAB. The behaviour of this protein on starch gel electrophoresis was closely similar to that of the basic carcinogen-binding protein present in the electrophoretic fraction " slow $h_2$ " which is deleted from primary hepatomata induced with DMAB (Sorof and Cohen, 1951; Sorof, Young and Ott, 1958; Sorof *et al.*, 1963). The present studies were initiated to investigate, using immunochemical procedures, the distribution of the basic azo-dye-binding liver protein in primary and transplanted rat hepatomata. These procedures provide a rapid and semi-quantitative assessment of the concentration of the protein in various tissue fractions which may be compared with data obtained from electrophoretic studies (Sorof *et al.*, 1963). Furthermore, the use of immunochemical techniques permits a study of the relationship of the azo-dye-binding protein to cell sap components previously found to be deleted from rat hepatomata (Baldwin, 1964; Baldwin and Barker, 1967*a*).

### MATERIALS AND METHODS

### *Rats*

Rats of an inbred Wistar strain were used for all tests and were maintained on a standard cubed diet (MRC 41B) with water *ad libitum.*

### *Tumour induction*

Hepatomata were induced in rats of both sexes by continuous oral administration of 4-dimethylaminoazobenzene (DMAB), 3′-methyl-4-dimethylaminoazobenzene (3′-methyl-DMAB), 2-acetylaminofluorene (AAF) and diethylnitro-

* Present address: Department of Medicine, Cambridge University.

samine (DENA), as previously described (Baldwin, 1964; Baldwin and Barker, 1967*a*). Tumours were transplanted subcutaneously into syngeneic rats of the same sex as the primary host.

### *Sub-cellular Fractionation of Tissues*

Rats were killed with ether or by cervical dislocation. Livers were perfused immediately with ice-cold 0·15 M NaCl followed by 0·44 M sucrose; other tissues and tumours were excised and rinsed in 0·44 M sucrose. All tissue samples were homogenized in 0·44 M sucrose (2 ml./g. wet weight of tissue) and microsomal and cell sap fractions obtained by differential centrifugation (Baldwin, 1964).

*Cell sap.*—Normal tissue fractions and tumour fractions were used immediately or stored at −20° C.

*Microsomes.*—Fractions from normal liver were solubilized in 0·4 per cent sodium deoxycholate in 0·25 M sucrose such that final solutions contained approximately 5–6 mg. protein/ml. (Baldwin, 1964).

*Nuclear sap.*—Livers, freshly perfused with saline and 0·44 M sucrose, were finely minced and homogenized in 2·1 M sucrose (2 ml./g. wet weight of tissue). Nuclei were isolated and extracts prepared in 0·12 M NaCl containing 0·01 M sodium phosphate pH 7·4, essentially as described by Bakay and Sorof (1964). Soluble nuclear extracts (nuclear sap) thus obtained were concentrated to approximately 10 mg. protein/ml. by dialysis against 25 per cent Ficoll (Pharmacia, Uppsala, Sweden) in buffered saline, pH 7·4.

### *Isolation of Basic Azo-Dye-Binding Protein*

Three- to four-month-old rats received 50 mg. DMAB in 2 ml. corn oil by intraperitoneal injection and were killed 16 hours later. Livers were perfused and homogenized in 0·25 M sucrose as described above. Subcellular particles were removed by centrifugation at 105,000 ***g*** for 2 hours. Basic azo-dye-binding protein was thereafter isolated from the resulting soluble cell supernatant (cell sap) as described by Ketterer, Ross-Mansell and Whitehead (1967). The final product was stored at a concentration of 3·65 mg. protein/ml. in buffered saline (0·15 M NaCl, 0·01 M Na phosphate, pH 7·4) at −20° C.

Tritium-labelled basic azo-dye-binding protein was similarly isolated from the livers of rats receiving 50 mg. tritiated 4-dimethylaminoazobenzene ($^{3}$H-DMAB, specific radioactivity, 0·40 mCi./mg.) labelled in the primed ring (Roberts and Warwick, 1966), by intraperitoneal injection in 2 ml. of corn oil. The final product was stored as above at a concentration of 0·8 mg. protein/ml.

For rabbit immunization, a basic preparation without bound azo-dye was isolated by an identical procedure from the livers of normal rats and stored at a concentration of 0·4 mg. protein/ml.

### *Preparation of Antisera*

Rabbit antisera against normal liver cell sap fractions were prepared as previously described (Baldwin, 1964).

For immunization against normal rat liver basic protein, aliquots of the preparation containing 0·4 mg. protein/ml. in buffered saline, pH 7·4, were emulsified with an equivalent volume of Freund's adjuvant (complete) and injected intramuscularly into adult rabbits. The immunization schedule consisted of 3 fortnightly injections of 2 ml. Freund's adjuvant mixture, 1 ml. being administered into each hind leg. Antisera were collected 3 weeks after the final injection, pooled and stored at $-20°$ C. with merthiolate added to a concentration of 0·01 per cent.

*Immunochemical Procedures*

Double diffusion analyses were carried out in 1 per cent agar gel in buffered saline (0·15 M NaCl, 0·01 M Na phosphate, pH 7·4) containing 0·2 per cent sodium azide as preservative. Basic azo-dye-binding protein was tested in buffered saline pH 7·4. Cell sap fractions, microsomal preparations and nuclear sap fractions were tested directly in 0·44 M sucrose, 0·4 per cent sodium deoxycholate in 0·25 M sucrose, and buffered saline, pH 7·4, respectively. Diffusion wells were filled once with each reagent and the sealed plates were incubated at room temperature and thence at 2° C.

In order to demonstrate basic azo-dye-binding protein in tissue fractions, samples were always cross-reacted in immunodiffusion tests with the purified basic azoprotein (usually 0·18 mg./ml.). For semi-quantitative estimations of the basic azo-dye-binding protein in tissue fractions, samples were initially adjusted to standard protein concentration (5 or 10 mg./ml.), and serial dilutions titrated to an end-point at which a precipitation reaction was just visible.

Under these conditions, patterns were normally fully developed within 2 to 3 days.

*Immunoelectrophoresis*

Immunoelectrophoresis was carried out on $20 \times 9·5$ cm. glass plates coated with a 2 mm. layer of 1 per cent Ionagar No. 2 (Oxoid, London) in veronal buffer pH 8·6, $\mu$ 0·025. Before analysis, cell sap fractions from normal liver were equilibrated with veronal buffer, pH 8·6, $\mu$ 0·05 and prepared for immunoelectrophoresis as previously described (Baldwin, 1964). Electrophoretic separations were performed at 2° C. employing a potential gradient of 4 volts/cm. for 3 hours. Under these conditions, a current of approximately 16 mA was required for each plate. Following electrophoresis, immunodiffusion patterns were developed at 2° C. for 2 to 3 days.

*Radioimmunoassay of Tritium-labelled Basic Azo-Dye-Binding Protein*

Conditions of slight antibody excess were initially determined by titration of the rabbit anti-basic protein antiserum against serial dilutions of a non-radioactive preparation of basic azoprotein.

Aliquots (0·2 ml.) of tritium-labelled basic azoprotein (0·082 mg., total radioactivity, $1·2 \times 10^4$ counts/min.) were incubated with periodic shaking for 3 days at 2° C. with aliquots (0·1 ml.) of the rabbit antiserum, or, in controls, with normal rabbit serum. Precipitates were separated by centrifugation (1000 $g$ for 10 minutes), washed twice in cold buffered saline (0·15 M NaCl, 0·01 M Na phosphate, pH 7·4) and assayed for radioactivity as described below. The supernatants were treated with a further volume (0·02 ml.) of the rabbit sera and any further precipitates washed and assayed together with the remaining supernatants as described.

### *Assay of Radioactive Samples*

Radioactive samples were assayed with a Packard Tricarb Liquid Scintillation Spectrophotometer (model 3375). All assays represent counts above background corrected for quenching by the channels ratio method (Herberg, 1965). Tritium-labelled basic azoprotein in aqueous solution was counted on glass-fibre discs as described by Davies and Cocking (1966). Antigen-antibody precipitates were assayed similarly after solubilization in 0·1 N sodium hydroxide.

### *Chemical Analysis*

Protein was determined by the Lowry technique (Lowry, Rosebrough, Farr and Randall, 1951) using bovine serum albumin as the primary standard.

### RESULTS

*Relationship of basic azo-dye-binding protein to normal liver cell sap protein.*

The relationship of the basic azo-dye-binding protein to normal liver cell sap proteins shown previously to be deleted from primary DMAB-induced hepatomata (Baldwin, 1964) was initially investigated. Of 11 rabbit antisera prepared against normal liver cell sap, 3 gave rise to a single precipitin line with the purified basic azoprotein (Fig. 1), which cross-reacted with a component of normal liver cell sap. It was found that the basic azoprotein (AZ) which was detectable in trace amounts in some tumour cell sap fractions (TCS) was not identifiable with other normal liver cell sap proteins (NCS) shown to be absent from primary hepatomata. In general, however, the multiplicity of reactions between the rabbit anti-normal liver cell sap (anti-NCS) and the normal liver cell sap fractions (NCS) did not always permit unequivocal identification of the basic azoprotein. More definitive distribution studies of the basic azoprotein in normal rat tissues and tumours were conducted using a specific rabbit anti-serum prepared against a basic protein fraction isolated without bound dye from the livers of normal, untreated rats.

*Immunochemical characterization of basic azo-dye-binding protein*

*Immunodiffusion.*—The rabbit antiserum prepared against the basic protein (anti-AZ) gave rise to a single precipitin line with purified basic azoprotein (AZ), which in turn cross-reacted with a similar preparation (K) supplied by Dr. Ketterer (Fig. 2), thus indicating the immunological identity of the two independently isolated samples. The sensitivity of the rabbit antiserum for detecting the basic azo-dye-binding protein in tissues was investigated by titration against serial dilutions of the protein (initial concentration, 3·65 mg. protein/ml.). An end-point was reached at a dilution of 1/1280 (Fig. 3), equivalent to 2·9 $\mu$g. protein/ml. The precipitation pattern obtained following reaction of the rabbit anti-basic protein antiserum with normal liver cell sap indicated the presence of antibody reacting with an additional component, unrelated to the basic azoprotein (Fig. 2). Whilst this component was frequently identified in tumours (see Fig. 7 and 9), as well as in normal tissues other than liver (Fig. 5), in no example studied did its presence interfere with the identification of the basic azoprotein.

*Immunoelectrophoresis.*—Basic azoprotein reacted as a single component with the rabbit antiserum (Fig. 4). Normal cell sap revealed two reactions of similar intensity, the faster of the two components closely corresponding to the basic azo-dye-binding protein.

*Radioimmunoassay.*—Basic azoprotein as the principal target of the rabbit antibody was confirmed by a series of radiochemical experiments. Tritium-labelled basic azoprotein was isolated from the liver cell sap of rats given $^{3}$H-DMAB. The level of covalently bound radioactivity in this preparation was equivalent to 80 $\mu$ mole DMAB per 100 g. protein. In comparison, the level of bound radioactivity in whole cell sap was equivalent to 15 $\mu$ mole DMAB per 100 g. protein. The specific radioactivity of the basic azo-dye-binding protein fraction was thus more than 5 times greater than that of whole cell sap. The tritium-labelled basic azo-protein gave rise to a single precipitation reaction and showed complete cross-reactivity with non-radioactive preparations.

### EXPLANATION OF PLATES

FIG. 1.—Agar gel precipitation reaction of basic azo-dye-binding protein and tissue cell sap fractions with antiserum prepared against normal liver cell sap (Anti-NCS).

NCS—Normal liver cell sap (10 mg./ml.).
TCS—DMAB-induced tumour cell sap (T1, 8·5 mg./ml., T2. 10·9 mg./ml.).
AZ—Basic azo-dye-binding protein (0·11 mg./ml.).

FIG. 2.—Cross-reactions in agar gel of basic azo-dye-binding protein (AZ, 0·8 mg./ml.), an identical preparation supplied by Dr. B. Ketterer (K, 0·2 mg./ml.), and a component of normal liver cell sap (10 mg./ml.) with antiserum prepared against the basic protein isolated from normal liver (Anti-AZ).

FIG. 3.—Dilution titration of basic azo-dye-binding protein (initial concentration, 3·65 mg./ml.) with rabbit anti-basic protein antiserum (Anti-AZ).

FIG. 4.—Immunoelectrophoresis of normal liver cell sap (NCS, 8·1 mg./ml.) and basic azo-dye-binding protein (AZ, 3·65 mg./ml.) using rabbit anti-basic protein antiserum (Anti-AZ).

FIG. 5.—Agar gel precipitation reactions of tissue cell sap fractions and basic azo-dye-binding protein (AZ, 0·18 mg./ml.) with rabbit anti-basic protein antiserum (Anti-AZ).

LU—lung (15·4 mg. protein/ml.).
KI—kidney (13·4 mg. protein/ml.).
SP—spleen (24·0 mg. protein/ml.).
BR—brain (4·9 mg. protein/ml.).

FIG. 6.—Agar gel precipitation reaction of basic azo-dye-binding protein (AZ, 0·11, mg./ml.), normal liver cell sap (NCS, 10 mg./ml.) and normal adult rat serum (NRS, undiluted) with anti-normal liver cell sap antiserum.

FIG. 7.—Agar gel precipitation reactions of primary DMAB-induced hepatoma cell sap fractions (T3CS and T4CS, 5·2 and 7·0 mg. protein/ml. respectively), normal liver cell sap (NCS, 7·0 mg./ml.) and basic azo-dye-binding protein (AZ, 0·365 mg./ml.) with rabbit anti-basic protein antiserum (Anti-AZ).

FIG. 8.—Agar gel precipitation reactions of non-tumour liver cell sap fractions (NTLi) and basic azo-dye-binding protein (AZ, 0·18 mg./ml.) with rabbit antibasic protein antiserum (Anti-AZ).
All tissue fractions, 10 mg. protein/ml.

FIG. 9.—Agar gel precipitation patterns of transplanted tumour cell sap fractions (all 10 mg./ml.) and basic azo-dye-binding protein (0·18 mg./ml.) with rabbit anti-basic protein antiserum (Anti-AZ).

AAF 5/8 Tumour AAF 5; generation 8.
DENA 1/6 Tumour DENA 1; generation 6.
D42/4 Tumour DMAB 42; generation 4.
D23/7 Tumour DMAB 23; generation 7.

1

2

3

Baldwin, Barker and Moore.

NCS
Anti AZ
AZ
Anti AZ

4

AZ
LU
SP
Anti AZ
KI
BR
AZ

5

AZ

NRS

Anti NCS

NCS

AZ

6

AZ
NCS
T3 CS
Anti AZ
T4
NCS
AZ

7

AZ
NT Li 1
NT Li 3
Anti AZ
NT Li 2
NT Li 4
AZ

8

AZ
AAF5 8
Dena
6
Anti AZ
D42 4
D23 7
AZ

9

Of the total radioactivity in the tritium-labelled basic azo-protein preparation, up to 69·5 per cent was precipitated in the presence of slight excess rabbit anti-basic protein antiserum (Table I). This represented over four-fifths of the total

TABLE I.—*Immunoprecipitation of Tritium-labelled Basic Azo-Dye-Binding Protein*

| Rabbit serum | Total radioactivity (counts/min.) in: Antigen-antibody precipitate | Supernatant | Overall recovery (per cent) |
|---|---|---|---|
| — | — | 11,950 | 100 |
| | | 11,850 | |
| Normal | 65 (0·5) | 9552 (81·6) | 82·1 |
| | 57 (0·5) | 8148 (68·7) | 69·2 |
| Anti-basic protein | 8231 (69·5) | 1435 (12·1) | 81·6 |
| | 7851 (66·3) | 1414 (11·9) | 78·2 |

Figures in parentheses represent percentage of total radioactivity in each fraction.

radioactivity recoverable from the test (81·6 per cent), the remaining 12·1 per cent of the radioactivity being present in the supernatant. Non-specific precipitation, assessed by treatment of the labelled protein with normal rabbit serum, accounted for no more than 0·5 per cent of the radioactivity. These experiments established that the basic azo-dye-binding protein can be effectively identified and estimated by reaction with rabbit anti-basic protein antibody.

*Distribution of basic azo-dye-binding protein in normal liver and other tissues.*

*Intracellular localization in liver.*—Cell sap, nuclear sap and solubilized microsome fractions (adjusted to 10 mg. protein/ml.) were titrated to their respective end-points against rabbit anti-basic protein anti-serum. Normal liver cell sap gave a dilution end point of 1/256 whereas titres obtained for nuclear sap (1/8) and microsomal preparations (1/16) were significantly lower.

These results indicate that, although present in nuclei and microsomes, the basic azo-dye-binding protein is predominantly a cytoplasmic protein.

*Normal rat tissues.*—Basic azo-dye-binding protein was detected in only one of the normal rat tissues other than liver, which were examined. Fig. 5 illustrates its presence in kidney (KI), and its absence from lung (LU), spleen (SP) and brain (BR) cell sap fractions. Kidney cell sap provides a typical example of a tissue fraction containing the component additional to basic azoprotein, which is reactive to the rabbit antiserum (Fig. 5). Clearly, its presence does not complicate the characterisation of the basic azoprotein.

The absence of basic azo-dye-binding protein from adult rat serum (NRS) was confirmed by failure of the basic azoprotein to cross-react with the component in serum reactive to rabbit anti-normal liver cell sap antiserum (Fig. 6).

*Distribution of basic azo-dye-binding protein in primary hepatomata*

In all, cell sap fractions from a total of 24 tumours induced by DMAB, 3′-methyl-DMAB, DENA and AAF were analysed for the presence of basic azo-dye-binding protein by comparison of the agar gel cross-reactions with the rabbit antiserum. From the typical result illustrated (Fig. 7), it may be seen that although absent from tumour cell sap fraction, T3CS, basic azoprotein is present in T4CS, since it forms a line of identity with purified basic azoprotein and a component of normal liver cell sap. In order to express the basic azo-dye-binding protein content of tumours relative to that of normal liver, cell sap fractions were titrated to an end-point against the rabbit antiserum. Basic azo-dye-binding protein was detectable in normal liver cell sap usually to a dilution end-point of 1/256, but in no primary tumour examined was the titre as high as this (Table II). Two DENA and two AAF-induced tumours gave titres of 1/64

TABLE II.—*Basic Azo-Dye-Binding Protein in Normal Rat Liver and Carcinogen-induced Primary Hepatomata*

| Tissue cell sap | Number of samples | Maximum cell sap dilution* at which basic protein detectable | | | | | | | | |
|---|---|---|---|---|---|---|---|---|---|---|
| | | Not detectable | $\frac{1}{2}$ | $\frac{1}{4}$ | $\frac{1}{8}$ | $\frac{1}{16}$ | $\frac{1}{32}$ | $\frac{1}{64}$ | $\frac{1}{128}$ | $\frac{1}{256}$ |
| Normal liver . . . | 4 | | | | | | | | 2 | 2 |
| Hepatomata induced by:— | | | | | | | | | | |
| DMAB . . . | 14 | 2 | 4 | 2 | 3 | 1 | 2 | | | |
| 3′-methyl-DMAB . | 6 | 1 | 1 | 2 | | | 2 | | | |
| AAF . . . | 2 | | | | | | | 2 | | |
| DENA . . . | 2 | | | | | | | 2 | | |

* Original cell sap concentration, 10 mg./ml.

corresponding to a concentration of 25 to 50 per cent of that in normal liver cell sap. The maximum titre obtained for tumours induced by DMAB and its 3′-methyl derivative was 1/32, indicating that the basic azo-dye-binding protein content did not exceed 25 per cent of that in normal liver cell sap, and was frequently very much lower even than this. However, in only 3 examples was the protein undetectable, the limit of detection being equivalent to 0·4 per cent of that in normal liver cell sap. Moreover, it is notable that in all examples of tumour cell sap fractions studied, deletion of the second component to which the rabbit antiserum was reactive was concomitant with the deletion of basic azo-dye-binding protein (Fig. 7 and 9).

In order to establish that the depletion of the basic protein in primary tumours was not due to non-specific dietary effects during carcinogen feeding, in a number of analyses, cell sap fractions prepared from apparently healthy liver taken from hepatoma-bearing rats were cross-reacted with purified basic azoprotein. All non-tumour liver fractions (NTLi) thus examined gave strong cross-reactions (Fig. 8), comparable with those of normal liver cell sap.

*Distribution of basic azo-dye-binding protein in transplanted hepatomata*

Cell sap fractions from tumours originally induced by DMAB, 3′-methyl-DMAB, DENA and AAF, and passaged in syngeneic hosts, were analysed for basic protein using the rabbit antiserum. In the typical example shown (Fig. 9) the basic protein was present only in the AAF-tumour analysed at generation 8 (AAF 5/8). The protein was undetectable in the DENA tumour at generation 6

(DENA 1/6), and the 2 tumours induced originally by DMAB examined at generations 4 and 7 (D42/4 and D23/7). In only 2 of 14 transplanted DMAB-hepatomata (D14 and D37) examined in this way, was the basic azo-dye-binding protein detectable (Table III). In the case of the transplanted DENA tumour, the protein was detectable at generation 4, but absent from generations 6 and 7, whereas in the case of those tumours induced originally with AAF, it was detected in 3 out of 4 tumours studied.

TABLE III.—*Basic Azo-Dye-Binding Protein in Transplanted Hepatomata*

| Tumour and transfer generations tested | Generations of transfer at which basic protein | |
|---|---|---|
| | Detectable | Undetectable |
| D8/4–40 | — | 4, 6, 21, 23, 40 |
| D14/5–8 | 5, 6, 7, 8 | — |
| D23/5–18 | — | 5, 7, 8, 17, 18 |
| D25/12 | — | 12 |
| D30/4–6 | — | 4, 6 |
| D31/4–12 | — | 4, 5, 12 |
| D32/1–2 | — | 1, 2 |
| D33/8–10 | — | 8, 9, 10 |
| D37/1 | 1 | — |
| D38/1 | — | 1 |
| D39/9 | — | 9 |
| D41/5 | — | 5 |
| D42/4 | — | 4 |
| D43/3 | — | 3 |
| AAF5/3–8 | 3, 8 | — |
| AAF28/3 | 3 | — |
| AAF29/5 | — | 5 |
| AAF35/3 | 3 | — |
| DENA1/4–7 | 4 | 6, 7 |

D, AAF, DENA: Tumours induced originally by 4-dimethylaminoazobenzene, 2-acetylaminofluorene and diethylnitrosamine.

### DISCUSSION

Immunodiffusion and immunoelectrophoretic analyses of a highly purified carcinogen-binding protein fraction (basic azoprotein) isolated from the livers of rats given DMAB with a specific rabbit antiserum prepared against the protein fraction isolated from normal liver, gave rise to single precipitation reactions thus demonstrating the immunochemical homogeneity of the protein. These observations are in accord with its chemical and physiochemical homogeneity reported by Ketterer, Ross-Mansell and Whitehead (1967), who also demonstrated that this protein corresponds to the azo-dye-binding protein present in the " slow $h_2$ " fraction of Sorof *et al.* (1963). It was further verified that the rabbit antiserum against the basic protein fraction reacted specifically with protein involved in carcinogen binding. Hence, tritium-labelled basic azoprotein isolated from the livers of rats given $^3$H-DMAB which showed complete immunological identity with azoprotein preparations from DMAB-treated liver was specifically precipitated by the rabbit antiserum (Table I). These observations thus establish the validity of the rabbit antiserum as an immunological reagent for the detection and estimation of this basic azo-dye-binding protein in tissue fractions.

By these means, it has been demonstrated that the concentration of this basic liver protein fraction specifically involved in binding of carcinogen metabolites is significantly reduced in primary and transplanted hepatomata. Furthermore, this protein is clearly distinguishable from other liver cell sap proteins shown previously to be deleted from tumour (Baldwin, 1964; Baldwin and Barker, 1967*a*).

Deletion of basic azoprotein was consistently demonstrated in the primary DMAB- and 3′-methyl-DMAB-induced hepatomata studied, although the extent varied between relatively wide limits (Table II). In a few tumours, no basic azoprotein was demonstrable, the limit of detection being equivalent to less than 0·4 per cent of that in normal liver, whereas in others the concentration approached 25 per cent of the normal liver value.

More marked deletion of the basic azo-dye-binding protein was observed in transplanted hepatomata, the only tumours in which it was detectable being hepatoma D14, where it was demonstrable up to the eighth transplant generation, and the first transplant generation of hepatoma D37. In all these studies, hepatomata were passaged in syngeneic hosts so that the more marked deletions of the basic protein in transplanted tumours are not likely to have occurred as a consequence of immunoselection. This is further emphasized by other studies demonstrating cell-membrane-associated tumour specific transplantation antigens which are still present after 20 generations of passage (Baldwin and Barker, 1967*b*). It is possible that the deletion of basic azo-dye-binding protein in transplanted hepatomata may be associated with the isolation of clones of tumour cells with enhanced proliferative properties (Abelev, 1965). Hence Reuber (1966) has postulated that primary liver lesions contain malignant cells of varying degrees of differentiation which differ in transplantability. In accordance with these concepts, it is of interest to note that in most cases the tumour growth potential, assessed either from the minimum cell inoculum necessary to produce progressive growth or from the tumour doubling time, is markedly increased after the first generation of transfer.

The increased growth potential of the DMAB-transplanted hepatomata concomitant, in most cases, with the deletion of basic azo-dye-binding protein, suggests the possibility that this protein may be involved in homeostatic control processes. But it should be emphasized that a whole series of cell sap, microsomal, and plasma membrane components are also deleted in these transplanted tumours (Baldwin and Barker, 1967*a*; Baldwin and Glaves, unpublished observations).

In the limited number of DENA- and AAF-induced hepatomata studied, the content of the basic protein was greater than that in DMAB-induced hepatomata, approaching 50 per cent of that in normal liver. Moreover, the basic protein was more frequently detected in the transplanted AAF-induced hepatomata (Table III). Hepatomata induced by DMAB were frequently poorly or moderately differentiated and grossly were firm grey or white and coarsely lobulated. In contrast, AAF- and DENA-induced hepatomata were usually more well differentiated and grossly were soft liver-coloured lesions. There were also marked differences in the growth properties of the hepatomata induced by different carcinogens. Thus the latent induction period of AAF-induced hepatomata (40–60 weeks) was considerably greater than that of either DMAB or DENA tumours (16–20 weeks). Furthermore, first transplant generations of DENA- and AAF-induced tumours had slower growth rates of up to 9 months compared to 4 months with DMAB-

induced tumours. In terms of growth properties, the transplanted AAF-induced hepatomata closely resemble the minimal deviation hepatomata (Morris, 1966) and in comparison, Sorof *et al.* (1965) have reported that the content of $h_2$ protein in these tumours is comparable with that in normal liver.

The present findings on deletion of a basic azo-dye-binding protein in carcinogen-induced hepatomata are in accord with the extensive data of Sorof and co-workers (Sorof and Cohen, 1951; Sorof, Young and Ott, 1958; Sorof *et al.*, 1963) which have established that a group of basic liver proteins (slow $h_2$) involved in the binding of metabolites of DMAB, is largely deleted from hepatomata induced by this carcinogen. In analogous immunochemical studies, Kitagawa *et al.* (1966) also demonstrated deletion from primary hepatomata of a liver microsomal component which interacts specifically with AAF.

Apart from the above examples, however, there is no evidence to suggest that the majority of the liver cell components deleted in hepatomata (Baldwin, 1964; Baldwin and Barker, 1967*a*) are involved in direct interaction with carcinogen metabolites in the pre-neoplastic stage. They may, for example, reflect mutational changes, the feasibility of which has been emphasized by the recent demonstration of *in vivo* interaction with nucleic acids, of DMAB (Roberts and Warwick, 1966), AAF (Sporn and Dingman, 1966) and DENA (Magee and Barnes, 1967).

Whilst the present findings indicate that the deletion of basic azo-dye-binding protein is not an essential requirement for neoplastic transformation, it may offer selective advantage to tumour cell clones in respect of their new growth potential. Such a concept is in accord with studies on the sensitivities of normal and neoplastic cells to carcinogens (Prehn, 1964; Vasiliev and Guelstein, 1967) which are considered to correlate with deficiencies of growth-controlling systems responsible for neoplastic cell properties.

### SUMMARY

The distribution of a basic azo-dye-binding protein isolated from 4-dimethylaminoazobenzene (DMAB)-treated rat liver in primary and transplanted DMAB-induced hepatomata has been studied using immunochemical techniques. Of 20 primary hepatomata examined, only 3 were found to lack the protein although in the remaining 17 the concentration was significantly reduced. In contrast, studies on 14 DMAB-induced transplanted hepatomata revealed the protein in 2 examples only.

Additionally, the protein was shown to be present in the limited number of primary hepatomata induced by 2-acetylaminofluorene and diethylnitrosamine which were examined, but was not always detected in subsequent transplants of these tumours.

The authors wish to thank Dr. B. Ketterer, Courtauld Institute of Biochemistry, Middlesex Hospital, London, for kindly providing a sample of basic azo-dye-binding protein and for helpful discussions in the preparation of this manuscript; Dr. J. J. Roberts, Chester Beatty Research Institute, London, for the gift of tritiated 4-dimethylaminoazobenzene, and Mrs. M. E. Marshall for skilled technical assistance.

This work was supported by a block grant from the British Empire Cancer Campaign for Research.

### REFERENCES

ABELEV, G. I.—(1965) *Prog. exp. Tumor Res.*, **7**, 104.
BAKAY, B. AND SOROF, S.—(1964) *Cancer Res.*, **24**, 1814.
BALDWIN, R. W.—(1964) *Br. J. Cancer*, **18**, 285.
BALDWIN, R. W. AND BARKER, C. R.—(1967*a*) *Nature, Lond.*, **214**, 292.—(1967b) *Int. J. Cancer*, **2**, 355.
DAVIES, J. W. AND COCKING, E. C.—(1966) *Biochim. biophys. Acta.*, **115**, 511.
HERBERG, R. J.—(1965) *Packard Technical Bulletin* No. 15.
KETTERER, B., ROSS-MANSELL, P. AND WHITEHEAD, J. K.—(1967) *Biochem. J.*, **103**, 316.
KITAGAWA, M., TANIGAKI, N., YAGI, Y., PLANINSEK, J. AND PRESSMAN, D.—(1966) *Cancer Res.*, **26**, 752.
LOWRY, O. H., ROSEBROUGH, N. J., FARR, A. L. AND RANDALL, R. J.—(1951) *J. biol. Chem.*, **193**, 265.
MAGEE, P. N. AND BARNES, J. M.—(1967) *Adv. Cancer. Res.*, **10**, 163.
MILLER, E. C. AND MILLER, J. A.—(1966) *Pharmac. Rev.*, **18**, 805.
MORRIS, H. P.—(1966) In 'Biological and Biochemical Evaluation of Malignancy in Experimental Hepatomas ', *Gann*, Monograph 1, p. 1.
PREHN, R. T.—(1964) *J. natn. Cancer Inst.*, **32**, 1.
REUBER, M. D.—(1966) In ' Biological and Biochemical Evaluation of Malignancy in Experimental Hepatomas ', *Gann*, Monograph 1, p. 43.
ROBERTS, J. J. AND WARWICK, G. P.—(1966) *Int. J. Cancer*, **1**, 179.
SOROF, S. AND COHEN, P. P.—(1951) *Cancer Res.*, **11**, 376.
SOROF, S., YOUNG, E., COFFEY, C. AND MORRIS, H. P.—(1965) *Proc. Am. Ass. Cancer Res.*, **6**, 60.
SOROF, S., YOUNG, E. M., MCCUE, M. M. AND FETTERMAN, P. L.—(1963) *Cancer Res.*, **23**, 864.
SOROF, S., YOUNG, E. M. AND OTT, M. G.—(1958) *Cancer Res.*, **18**, 33.
SPORN, M. B. AND DINGMAN, C. W.—(1966) *Nature, Lond.*, **210**, 531.
VASILIEV, J. M. AND GUELSTEIN, V. I.—(1967) In ' Control of Cellular Growth in Adult Organisms ', Edited by H. Teir and T. Rytömaa, London (Academic Press), p. 319.